# Overview of the clinical efficacy and safety of eldecalcitol for the treatment of osteoporosis

**DOI:** 10.1007/s11657-022-01071-3

**Published:** 2022-05-05

**Authors:** Lijia Cui, Weibo Xia, Chuan Yu, Shuangshuang Dong, Yu Pei

**Affiliations:** 1 Department of Endocrinology, Peking Union Medical College Hospital, Chinese Academy of Medical Sciences and Peking Union Medical College, Beijing, 100005 China; 2 Chugai Pharma China CO., LTD, Shanghai, 200021 China; 3 Department of Endocrinology, First Medical Center, General Hospital of the People’s Liberation Army of China, Beijing, 100039 China

**Keywords:** Eldecalcitol, Bone mineral density, Osteoporosis, Fracture, Systematic review

## Abstract

**Summary:**

Eldecalcitol (ELD) is a new oral analog of the active form of vitamin D with anti-resorptive properties. We conducted a meta-analysis to investigate the efficacy and safety of ELD in osteoporosis. Compared with alfacalcidol, ELD significantly lowered vertebral facture risk, increased bone mineral density, but also had a higher risk of hypercalciuria.

**Purpose:**

This study aimed to investigate the efficacy and safety of eldecalcitol (ELD) in osteoporosis by examining fracture rates, bone mineral density (BMD), bone turnover markers, and adverse events as outcomes.

**Methods:**

PubMed, EMBASE, and Cochrane Library were searched up to July 20, 2020, to identify eligible randomized controlled trials. The odds ratio (OR) or weighted mean difference (WMD) with 95% confidence interval was calculated by the random-effects model.

**Results:**

ELD significantly increased lumbar BMD (WMD: 2.80; 95% CI: 1.60, 4.00; *P* < 0.001, 2 studies involved), total hip BMD (WMD: 2.11; 95% CI: 0.68, 3.55; *P* = 0.004, 2 studies involved), and femoral neck BMD (WMD: 1.78; 95% CI: 0.76, 2.79; *P* = 0.001, 1 study involved) compared with alfacalcidol. Moreover, ELD caused a significantly lower rate of vertebral fracture (OR: 0.52; 95% CI: 0.29–0.95; *P* = 0.034, 2 studies involved) than alfacalcidol, but did not lower the rate of non-vertebral facture (OR: 0.44; 95% CI: 0.06–3.05; *P* = 0.405, 2 studies involved) compared with alfacalcidol. ELD significantly reduced the percentage change in bone-specific alkaline phosphatase (WMD: − 15.40; 95% CI: − 20.30, − 10.60; *P* < 0.001, 1 study involved) and serum type I collagen C-telopeptide (WMD: − 38.50; 95% CI: − 50.00, − 27.10; *P* < 0.001, 1 study involved) as compared with alfacalcidol. ELD was also associated with higher risk of hypercalciuria compared with alfacalcidol (OR: 1.64; 95% CI: 1.22, 2.20; *P* = 0.001, 2 studies involved).

**Conclusions:**

This systematic review indicated that ELD was superior than alfacalcidol for improving vertebral fracture risk and BMD. Further large-scale trials should be conducted to verify the long-term effects and safety of ELD in osteoporosis.

**Prospero registration number:**

CRD42020147518.

**Supplementary Information:**

The online version contains supplementary material available at 10.1007/s11657-022-01071-3.

## Introduction

The deficiency of vitamin D is common in elderly men and postmenopausal women [1], while it is associated with a decrease in calcium absorption, bone mineral density (BMD) level and muscle strength, and an increase of fracture risk from falling [2]. Therefore, it is important to provide adequate vitamin D supplementation, especially in elderly men and postmenopausal women. Vitamin D is metabolized to 25-hydroxyvitamin D [25(OH)D], and transformed into 1α,25(OH)_2_D in the kidney [3]. The active form of vitamin D may show ideal clinical benefits in elderly patients, and patients with renal insufficiency and alpha 1 hydroxylase deficiency.

Eldecalcitol (ELD) is a new oral analog of 1α,25(OH)_2_D with a hydroxypropyloxy residue introduced at the 2β position, and has been approved for osteoporosis treatment. Several clinical studies have indicated that ELD increased lumbar BMD, inhibited bone turnover markers (BTMs), and reduced the vertebral fracture rate [4]. However, previous systemic reviews on ELD focused mainly on serum and urinary parameters, and clinical endpoints such as fractures, but did not systematically examine the adverse events of ELD or include several recently published randomized controlled trials (RCTs) on ELD [5–7]. Therefore, we performed a systematic review of ELD, using up-to-date literature, to assess the efficacy and safety of ELD for the treatment of osteoporosis, using BMD, BTMs, fracture rates, calcium, phosphorus and vitamin D metabolites, and adverse events as the investigated outcomes.

## Material and methods

### Data sources, search strategy, and selection criteria

This study was conducted according to the Preferred Reporting Items for Systematic Reviews and Meta-Analysis (PRISMA) statement [8]. The protocol of this study was registered in the international prospective register of systematic reviews: the registration number was CRD42020147518. Studies on the effectiveness of ELD for the treatment of osteoporosis published in English were included in the analysis. We systematically searched PubMed, EMBASE, and Cochrane Library up to July 20, 2020, using the following search criteria: (English [Language]) AND (eldecalcitol [Mesh] OR eldecalcitol [Title/Abstract] OR ED-71 [Title/Abstract]) AND (human [Species]). Manual searches were performed from the reference lists to identify additional relevant studies.

The literature search and the selection of studies were conducted by two researchers independently and any conflict was settled by group discussion until a consensus was reached. A study was eligible if it met the following inclusion criteria: (1) patients: patients with osteoporosis; (2) intervention: ELD, irrespective of combination with other treatment strategies; (3) control: placebo or other anti-osteoporosis treatment strategies; (4) outcomes: BMD, BTMs, fracture rates, calcium, phosphorus, vitamin D metabolites, and adverse events; and (5) study design: randomized controlled trials (RCTs).

### Data collection and quality assessment

The following details were extracted from each included study: first author’s surname, publication year, location, sample size, sex ratio, mean age, BMI, interventions, co-interventions, baseline vitamin D status, and follow-up period. The outcomes of BMD, bone turnover markers (BTMs), fracture rates, calcium, phosphorus, vitamin D metabolites, and adverse events were analyzed. We assessed the methodological quality of the included RCTs using a risk of bias approach according to the methods described by the Cochrane Collaboration, which included seven specified domains (random sequence generation, allocation concealment, blinding of participants and personnel, blinding of outcome assessment, incomplete outcome data, selective reporting, and other) [9]. Data extraction and quality assessment were conducted independently by two researchers, and inconsistency between researchers was resolved by group discussion until a consensus was reached.

### Statistical analysis

Quantitative analysis was based on extracted data after data transform. The extracted data were classified as dichotomous or continuous variables. The occurred events and sample size in intervention and control groups were abstracted as dichotomous variables, and the odds ratio (OR) with 95% confidence intervals (CIs) was calculated. Moreover, the continuous variables were abstracted as mean, standard deviation, and sample size in intervention and control groups; then, the weighted mean difference (WMD) with 95% CIs were calculated. All of the pooled results were performed using the random-effects model [10]. Heterogeneity across included trials was assessed by using *I*^*2*^ and Q statistics, and *p*-value for heterogeneity less than 0.10 was considered as significant heterogeneity [11, 12]. The *p*-values for pooled results were reported as two-sided, and *p*-values less than 0.05 were considered statistically significant. Stata (version 14.0) and Review Manager (version 5.3) were used for the meta-analysis.

## Results

### Study selection

**Fig. 1:**
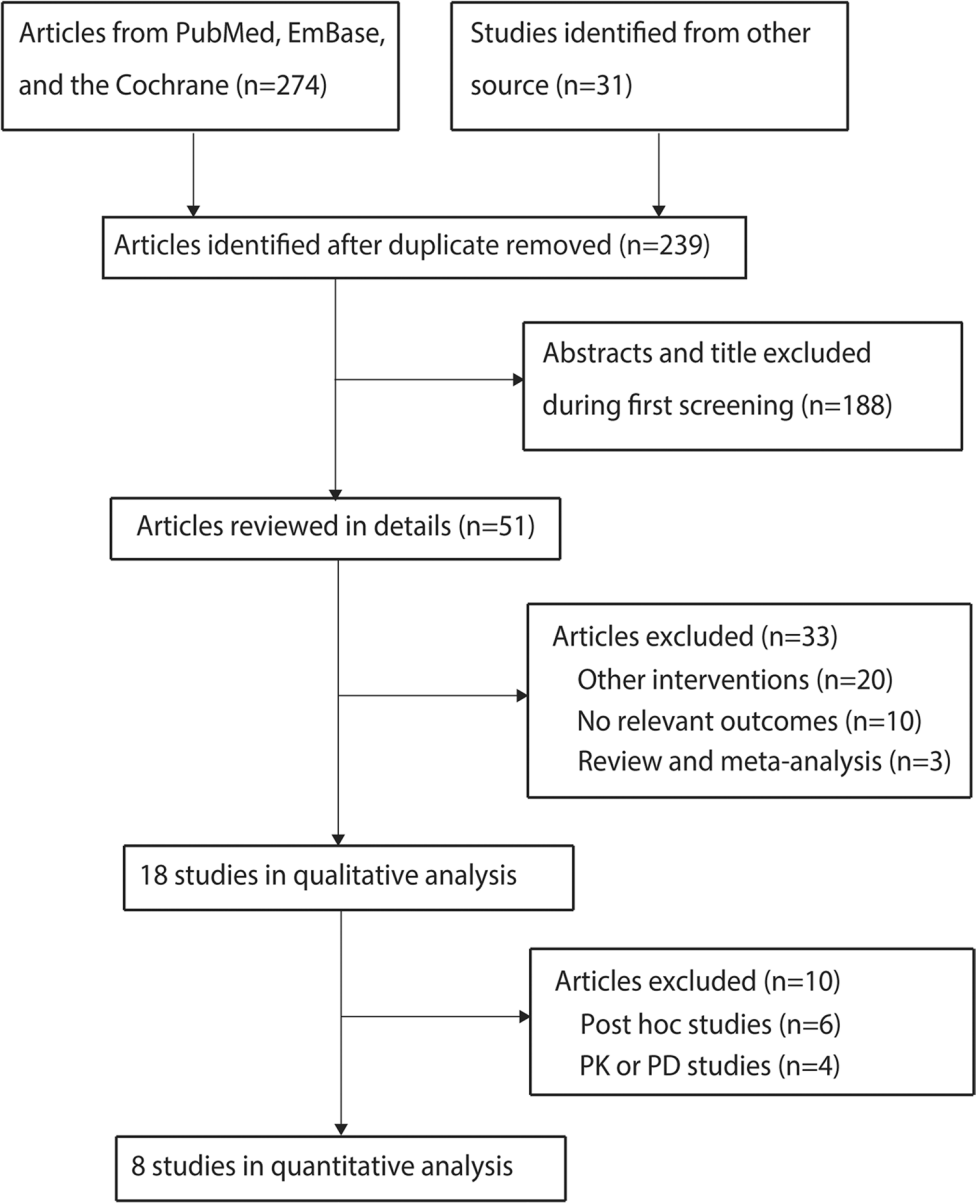
Flow diagram of selection process of the studies

The literature search and the selection process are summarized in Fig. 1. We identified 305 articles in the public electronic database search, and 66 studies were excluded due to redundancy in topics. The remaining 239 studies were retrieved for further evaluation, and 188 studies were excluded after screening the title and abstract. A total of 51 studies were obtained for full-text evaluations, and 33 studies were excluded because of other intervention (*n* = 20), no relevant outcomes (*n* = 10), and review or meta-analysis (*n* = 3). After this, 10 studies were excluded owing to post hoc studies or PK or PD studies. Finally, 8 eligible studies of high quality were selected for quantitative analysis [13–20].

### Characteristics of included studies

**Fig. 2:**
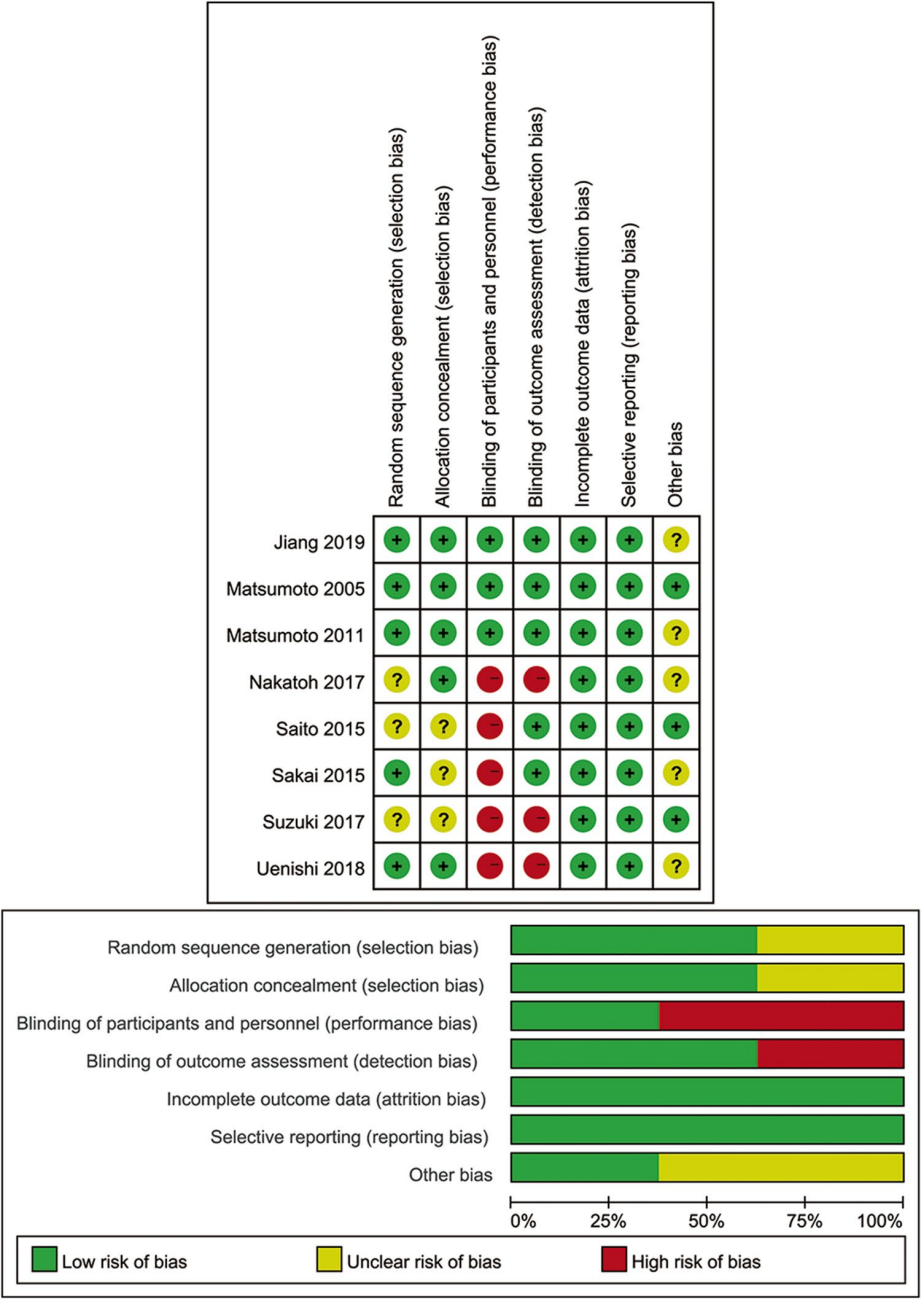
Risk-of-bias assessments for included trials

Among the selected eight studies, 2204 individuals were included in total, with a mean age range of 64.9–83.0 years. One study included patients with high body mass index (BMI > 24 kg/m^2^) [16], and the remaining seven studies included patients with normal BMI (BMI < 24 kg/m^2^) [13–15, 17–20]. Seven of the RCTs were conducted in Japan, and one was conducted in China. The follow-up period ranged from 4 weeks to 36 months. In addition to ELD, alfacalcidol, vitamin D_3_, vitamin D plus calcium, and placebo were used as interventions. Furthermore, the co-intervention drugs included vitamin D_3_, alendronate, minodronate, raloxifene, and denosumab (Table 1). Among the eight included RCTs, three studies blinding of participants and personnel, and 5 studies blinding of outcome assessment. Moreover, 3 studies did not give the details describe of random sequence generation and allocation concealment. Furthermore, a total of 5 studies did not describe the potential source of other bias (Fig. 2).

**Table 1:** Baseline characteristics of included studies

Study	Country	Sample size	Number of participants (men/women)	Age (years), mean (SD)	BMI (kg/m^2^), mean (SD)	Intervention (dosage)	Co-intervention	Baseline vitamin D status (ng/ml)	Follow-up duration
Matsumoto 2005 [13]	Japan	55	NA	67.9 (7.7)	21.8 (3.4)	Eldecalcitol (0.5 µg/day)	Vitamin D3	17.2	12 months
		55	NA	66.3 (6.6)	21.6 (3.0)	Eldecalcitol (0.75 µg/day)		16.3	
		56	NA	66.8 (7.6)	22.1 (3.0)	Eldecalcitol (1 µg/day)		17.9	
		53	NA	68.0 (7.7)	22.7 (2.9)	Placebo		17.3	
Matsumoto 2011 [14]	Japan	528	9/519	72.2 (6.60)	22.2 (3.19)	Eldecalcitol (0.75 µg/day)	Vitamin D3	27.6	36 months
		526	15/521	72.1 (6.64)	22.3 (3.20)	Alfacalcidol (1.0 µg/day)		27.1	
Sakai 2015 [15]	Japan	110	1/109	71.5 (7.3)	22.3 (3.1)	Eldecalcitol (0.75 µg/day)	Alendronate	18.7	48 weeks
		109	4/105	71.6 (6.6)	21.7 (2.9)	Vitamin D 400 IU + calcium 610 mg daily		19.2	
Saito 2015 [16]	Japan	18	0/18	73 (6)	26.8 (3.9)	Eldecalcitol (0.75 µg/day)	Alendronate	NA	6 months
		17	0/17	72 (4)	27.2 (4.8)	Control		NA	
Nakatoh 2018 [17]	Japan	38	0/38	83.0 (5.4)	21.4 (3.2)	Eldecalcitol (0.75 µg/day)		NA	48 weeks
		41	0/41	81.6 (5.0)	21.6 (3.4)	Alfacalcidol (1.0 µg/day)	Minodronate	NA	
		42	0/42	82.7 (5.5)	21.7 (4.3)	Alfacalcidol (1.0 µg/day)	Raloxifene	NA	
Suzuki 2017 [18]	Japan	24	0/24	75.6 (1.4)	21.1 (0.8)	Eldecalcitol (0.75 µg/day)	Denosumab	NA	12 months
		26	0/26	75.7 (1.4)	21.2 (0.7)	Native vitamin D		NA	
Uenishi 2018 [19]	Japan	9	0/9	74.2 (2.4)	NA	Eldecalcitol (0.75 µg/day)		24.7	4 weeks
		9	0/9	75.0 (3.0)	NA	Alfacalcidol(1.0 µg/day)		20.0	
		10	0/10	72.9 (3.1)	NA	Plain vitamin D3 (800 IU/day)		23.9	
		10	0/10	74.6 (3.0)	NA	Control		21.3	
Jiang 2019 [20]	China	128	2/126	66.0 (6.9)	22.6 (3.5)	Eldecalcitol (0.75 µg/day)		15.6	12 months
		121	5/116	64.9 (7.1)	22.7 (3.0)	Alfacalcidol (1.0 µg/day)		17.0	

*BMI* body mass index; *NA* not available

### Bone mineral density

**Fig. 3:**
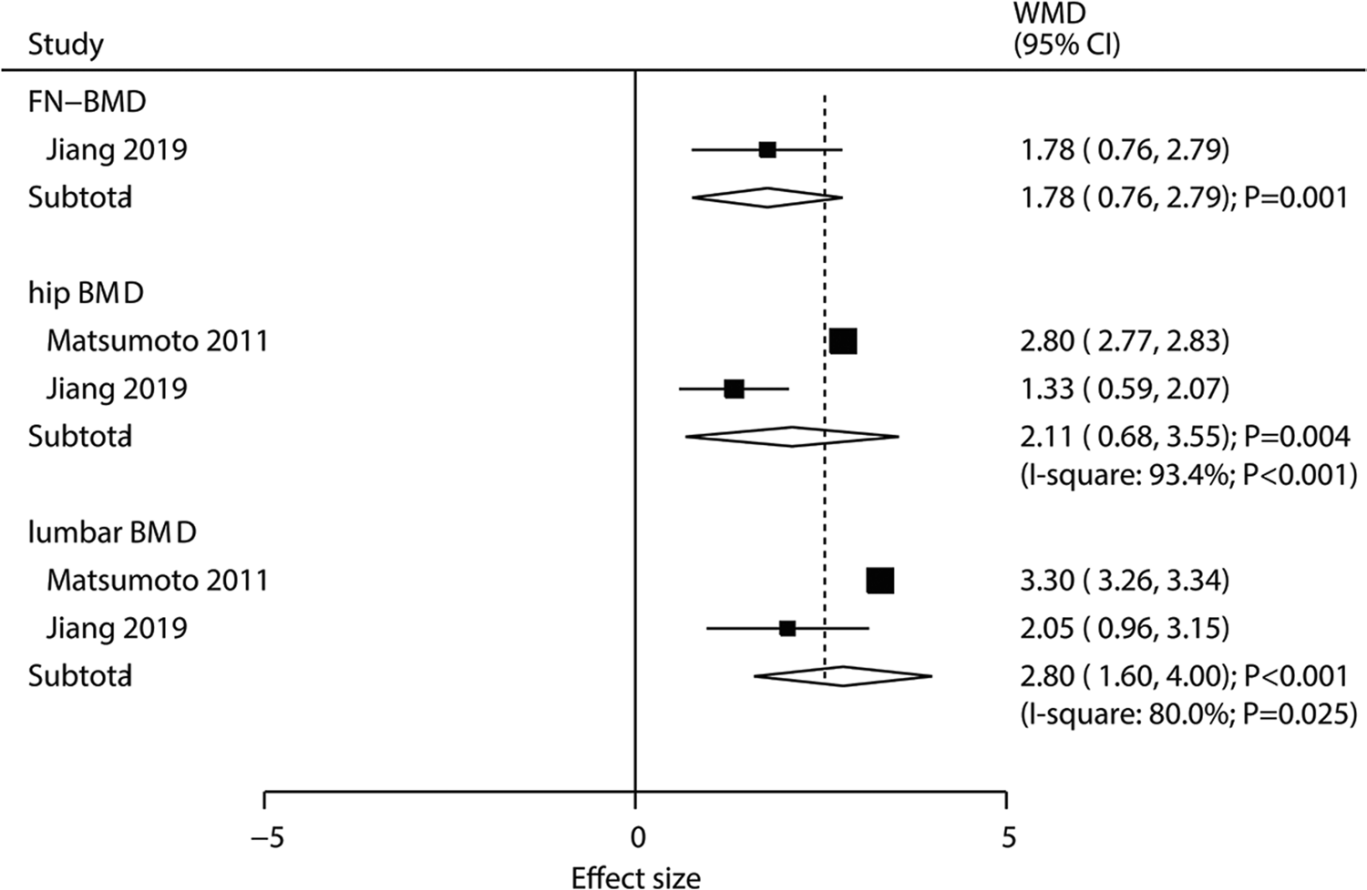
Random-effects meta-analysis of eldecalcitol compared with other active vitamin D on bone mineral density at various sites. BMD: bone mineral density; WMD: weighted mean difference; 95% CI: 95% confidence interval

Compared with placebo, treatment of ELD (1 μg/day) for 12 months significantly improved lumbar BMD by 3.1% and total hip BMD by 0.9% [13]. When compared with alfacalcidol, ELD (0.75 μg/day) was associated with an increased in the lumbar BMD (WMD: 2.80; 95% CI: 1.60, 4.00; *P* < 0.001; *I*^*2*^ = 80.0%; *P*_*heterogeneity*_ = 0.025) [14, 20], total hip BMD (WMD: 2.11; 95% CI: 0.68, 3.55; *P* = 0.004; *I*^*2*^ = 93.4%; *P*_*heterogeneity*_ < 0.001) [14, 20], and femoral neck BMD (WMD: 1.78; 95% CI: 0.76, 2.79; *P* = 0.001) (Fig. 3) [20]. The results of other comparisons and the BMD at other sites after intervention are presented in Supplementary Table 2, while no significant difference was detected.

### Fracture incidence

**Fig. 4:**
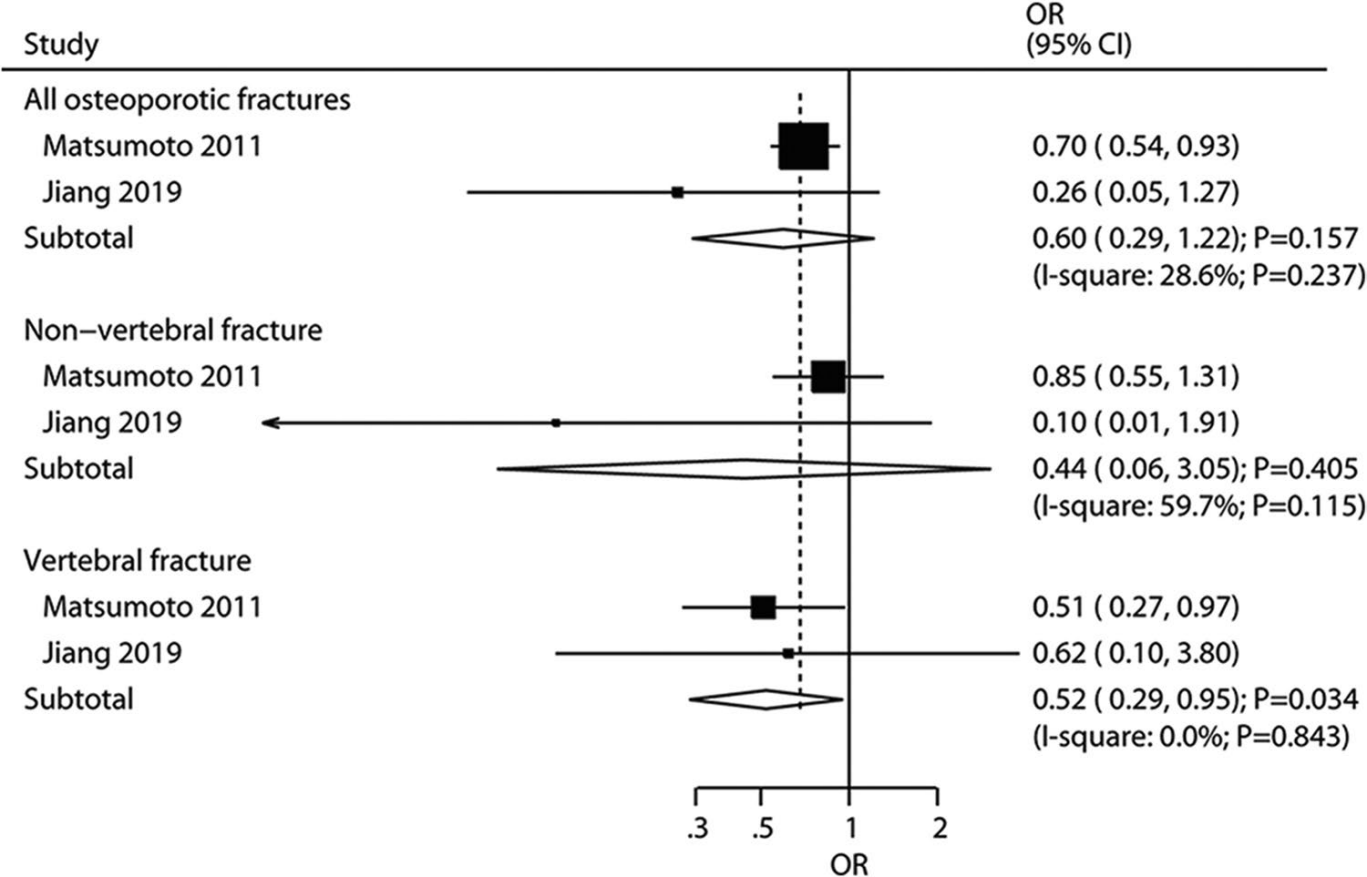
Random-effects meta-analysis of eldecalcitol compared with other active vitamin D on fracture rates. OR: odds ratio; 95% CI: 95% confidence interval

Compared with alfacalcidol, ELD caused a significantly lower risk of vertebral fracture (OR: 0.52; 95% CI: 0.29–0.95; *P* = 0.034; *I*^*2*^ = 0.0%; *P*_*heterogeneity*_ = 0.843) [14, 20], but did not lower the risk of non-vertebral fracture (OR: 0.44; 95% CI: 0.06–3.05; *P* = 0.405; *I*^*2*^ = 59.7%; *P*_*heterogeneity*_ = 0.115) (Fig. 4 and Supplementary Table 1) [14]. ELD was also associated with a lower risk of wrist fracture (OR: 0.29; 95% CI: 0.11, 0.77; *P* = 0.013) [21], and distal forearm fracture (OR: 0.28; 95% CI: 0.11, 0.69; *P* = 0.007), and had no significant effect on the risk of fractures in other parts of the body when compared with alfacalcidol or vitamin D plus calcium supplementation (Supplementary Table 1).

### Bone turnover markers

Compared with placebo, treatment of ELD (1.0 μg/day) for 3 months decreased urinary NTX by 24% [13]. When compared with alfacalcidol, ELD was associated with lower BALP (WMD: − 15.40; 95% CI: − 20.30, − 10.60; *P* < 0.001) [20] and serum type I collagen C-telopeptide (CTX-1) (WMD: − 38.50; 95% CI: − 50.00, − 27.10; *P* < 0.001) (Supplementary Table 2) [20]. In addition, a significant reduction in NTX was observed in ELD compared with vitamin D3 (WMD: − 9.30; 95% CI: − 17.03, − 1.57; *P* = 0.018) [19] and placebo (WMD: − 12.40; 95% CI: − 21.28, − 3.52; *P* = 0.006) [19]. Finally, ELD was associated with a higher TRACP − 5b level than minodronate (WMD: 84.00; 95% CI: 29.28, 138.72; *P* = 0.003) (Supplementary Table 2) [17].

### Calcium, phosphorus, and vitamin D metabolites

In terms of vitamin D metabolites, ELD (0.75 μg/day) was compared with alfacalcidol (1.0 μg/day), plain vitamin D_3_ (800 IU/day), minodronate (50 mg/28 days), or raloxifene (60 mg/day) and control in various clinical trials. ELD was associated with a lower serum 1,25(OH)2D level compared with alfacalcidol (WMD: − 72.30; 95% CI: − 76.60, − 68.00; *P* < 0.001) [14] and lower 25(OH)D level compared with plain vitamin D_3_ (WMD: − 10.00; 95% CI: − 17.52, − 2.48; *P* = 0.009) (Supplementary Table 2) [19].

The summary of effects of ELD on fractional calcium absorption (FCA), absorbed calcium (Abs), and urinary excretion of calcium (U-Ca) is presented in Supplementary Table 2. There were no significant differences between ELD and alfacalcidol in FCA (WMD: 3.50; 95% CI: − 3.40, 10.40; *P* = 0.320), Abs (WMD: 25.10; 95% CI: − 24.84, 75.04; *P* = 0.325), and U-Ca (WMD: − 33.00; 95% CI: − 100.76, 34.76; *P* = 0.340). However, compared with plain vitamin D_3_, ELD was associated with greater FCA (WMD: 9.00; 95% CI: 2.88, 15.12; *P* = 0.004) [19] and Abs (WMD: 65.10; 95% CI: 20.78, 109.42; *P* = 0.004) [19], but not U-Ca (WMD: 35.40; 95% CI:-27.99, 98.79; *P* = 0.274). Compared with placebo, ELD significantly increased the levels of FCA (WMD: 13.30; 95% CI: 8.27, 18.33; *P* < 0.001) [19], Abs (WMD: 96.20; 95% CI: 59.89, 132.51; *P* < 0.001) [19], and U-Ca (WMD: 87.00; 95% CI: 29.43, 144.57; *P* = 0.003) (Supplementary Table 2) [19].

### Adverse events

The adverse events of ELD are summarized in Supplementary Table 3. ELD was associated with higher risk of hypercalciuria (OR: 1.64; 95% CI: 1.22, 2.20; *P* = 0.001) and lower risks of constipation (OR: 0.64; 95% CI: 0.42, 0.98; *P* = 0.039) and exanthem (OR: 0.44; 95% CI: 0.23, 0.81; *P* = 0.011) compared with alfacalcidol. In addition, ELD was associated with a higher risk of ear and labyrinth disorders (OR: 8.47; 95% CI: 1.04, 68.92; *P* = 0.046) compared with vitamin D plus calcium supplementation.

## Discussion

Our study analyzed the clinical efficacy and safety of ELD in the treatment of osteoporosis by using systemic review and meta-analysis of prospective RCTs. ELD was associated with lowered rates of vertebral fracture, when compared with alfacalcidol. Moreover, ELD caused a significantly higher lumbar, total hip, and femoral neck BMD than alfacalcidol. ELD also significantly decreased BALP and serum type I collagen C-telopeptide (CTX-1) after treatment as compared with alfacalcidol. All these findings suggested that ELD was a better intervention for osteoporosis than alfacalcidol in elderly patients and postmenopausal women.

In a previous meta-analysis, ELD was found to improve the lumbar BMD, reduce NTX and BALP levels, and reduce the frequency of vertebral fractures, which was consistent with our results [4]. By reviewing the most up-to-date clinical trials of ELD published from 2017 to 2019, we evaluated the adverse events, and found that ELD did not increase any major adverse events compared with other interventions. However, it was worth noting that ELD is associated with a higher risk of hypercalciuria than alfacalcidol, and that it should be administered with caution in patients with urolithiasis.

When compared with alfacalcidol, ELD significantly increased the lumbar, total hip, and femoral neck BMD, and reduced the risk of vertebral fracture. These results were based on two trials with high quality [14, 20]. Moreover, ELD significantly reduced some BTMs, including BALP and CTX, as compared with alfacalcidol. A potential explanation was that ELD could better increase calcium absorption and urinary calcium excretion, and inhibit bone resorption compared with alfacalcidol [22, 23]. It was proposed that ELD had higher serum vitamin D-binding protein affinity and weaker binding affinity to vitamin D receptor than alfacalcidol, which therefore reduced its influence on serum parathyroid hormone [21].

The analysis of this study included seven studies performed on Japanese patients and one study performed on Chinese patients. Apart from differences in living habits, the characteristics and race were similar between Japanese and Chinese patients. There was no obvious difference found in ELD pharmacokinetics. It was suggested that ELD was well tolerated in Chinese patients at a dose range of 0.5 to 0.75 µg, which was similar to that reported for Japanese patients [24]. However, the pharmacological and clinical effects of ELD still need to be confirmed in patients of other races.

Tartrate-resistant acid phosphatase 5b (TRACP-5b) is a specific indicator of osteoclast activity and bone resorption. A study conducted by Iba et al. [25] suggested that ELD could significantly reduce TRACP-5b levels in postmenopausal women who have undergone long-term bisphosphonate treatment. Moreover, ELD could further decrease TRACP-5b level even if patients have received bisphosphonate for a long time. This result suggested that the mechanism of the inhibition of bone resorption could be different between bisphosphonate and ELD, and thus supported the combination of ELD and bisphosphonates in clinical use [26].

The use of an active form of vitamin D_3_ will further enrich the clinical treatment for elderly patients and patients with postmenopausal osteoporosis. In the included studies, the combination of drugs with different mechanisms was often used as a means of intervention. For example, denosumab, an activator targeting nuclear factor kappa B receptor which inhibited the activation of osteoclasts, was combined with ELD in the study design. A study of animal models also found that the combination of ELD and raloxifene increased bone strength, and BMD, and inhibited bone turnover in ovariectomized animal models [27]. With the emergence of more studies, head-to-head comparisons between different therapeutic strategies are likely to be the direction of further research.

This study has certain advantages. Previous systematic reviews on ELD only summarized the effect of ELD on BMD, bone turnover markers, and fracture rate [4, 5], but did not systematically illustrate potential adverse events of ELD. Moreover, they did not include several newly published RCTs on ELD [6, 7]. Our study is the most up-to-date systematic review and meta-analysis of ELD, which fully assessed the efficacy and safety of ELD for the treatment of osteoporosis, using BMD, BTMs, fracture rates, calcium, phosphorus, vitamin D metabolites, and adverse events as the investigated outcomes.

The systematic review has also limitations. First, pooled data at the trial level rather than individual data level restricted us from performing a more detailed relevant analysis. Second, this study was only based on a small number of studies owing to the limited number of high-quality RCTs on ELD published, and the results needed further large-scale RCT verification. Third, the use of background agents could have an impact on the efficacy of ELD. Fourth, as heterogeneity existed among the included studies, different co-intervention drugs and different individual characteristics could not be clearly defined and may have led to confounding effects.

This systemic review indicated that ELD significantly reduced vertebral fracture risk, increased BMD at various sites, lowered certain BTMs, and increased the risk of hypercalciuria. Further large-scale RCTs on ELD should be conducted to evaluate the long-term effects of ELD in Asian patients as well as in other races, and to explore the combined use of ELD with other osteoporosis treatment strategies.

## Supplementary Information

Below is the link to the electronic supplementary material.

Supplementary file1 (DOCX 37 KB)

## Data Availability

The datasets used and/or analyzed during the current study are available from the corresponding author on reasonable request.

## Abbreviations

ELD: Eldecalcitol
BMD: Bone mineral density
OR: Odds ratio
WMD: Weighted mean difference
CI: Confidence interval
RCTs: Randomized controlled trials
PRISMA: Preferred Reporting Items for Systematic Reviews and Meta-Analysis
BTMs: Bone turnover markers
CTX-1: Type I collagen C-telopeptide
FCA: Fractional calcium absorption
Abs: Absorbed calcium
PTH: Parathyroid hormone
FGF23: Fibroblast growth factor 23
TRACP-5b: Tartrate-resistant acid phosphatase 5b
