# Supplementary material for: Overview of the clinical efficacy and safety of eldecalcitol for the treatment of osteoporosis
Source: Arch Osteoporos. 2022 May 5;17(1):74. doi: 10.1007/s11657-022-01071-3 (PMC9072485; doi:10.1007/s11657-022-01071-3)
Supplement: Supplementary file 1 — Supplementary file1 (DOCX 37 KB) [file 11657_2022_1071_MOESM1_ESM.docx]

Supplementary Materials

**Supplementary Table 1. Summary of fracture outcomes at various sites by different interventions**

| Outcomes | Interventions | Included study | OR (95% CI) | P value |
| --- | --- | --- | --- | --- |
| Major three sites of non-vertebral (humerus, wrist and hip) | ELD (0.75 μg/day) versus ALF (1.0 μg/day) | Matsumoto 2011 | 0.51 (0.23-1.03) | 0.062 |
| Wrist fracture | ELD (0.75 μg/day) versus ALF (1.0 μg/day) | Matsumoto 2011 | 0.29 (0.11-0.77) | 0.013 |
| Ribs fracture | ELD (0.75 μg/day) versus ALF (1.0 μg/day) | Matsumoto 2011 | 0.56 (0.23-1.33) | 0.195 |
| Pelvis fracture | ELD (0.75 μg/day) versus ALF (1.0 μg/day) | Matsumoto 2011 | 2.95 (0.31-28.34) | 0.348 |
| Humerus fracture | ELD (0.75 μg/day) versus ALF (1.0 μg/day) | Matsumoto 2011 | 1.92 (0.17-21.22) | 0.596 |
| Clavicle, scapula, sternum fracture | ELD (0.75 μg/day) versus ALF (1.0 μg/day) | Matsumoto 2011 | 0.33 (0.03-3.20) | 0.352 |
| Hip fracture | ELD (0.75 μg/day) versus ALF (1.0 μg/day) | Matsumoto 2011 | 1.41 (0.45-4.44) | 0.556 |
| Tibia fracture | ELD (0.75 μg/day) versus ALF (1.0 μg/day) | Matsumoto 2011 | 0.20 (0.02-1.69) | 0.155 |
| Distal forearm fracture | ELD (0.75 μg/day) versus ALF (1.0 μg/day) | Matsumoto 2011 | 0.28 (0.11-0.69) | 0.007 |
| All osteoporotic fractures | ELD (0.75 μg/day) versus vitamin D 400 IU/day + Ca 610 mg/day | Sakai 2015 | 0.67 (0.25-1.82) | 0.432 |
|  | ELD (0.75 μg/day) versus ALF (1.0 μg/day) | Matsumoto 2011, Jiang 2019 | 0.60 (0.29-1.22) | 0.157 |
| Vertebral fracture | ELD (0.75 μg/day) versus vitamin D 400 IU/day + Ca 610 mg/day | Sakai 2015 | 1.41 (0.43-4.61) | 0.570 |
|  | ELD (0.75 μg/day) versus ALF (1.0 μg/day) | Matsumoto 2011, Jiang 2019 | 0.52 (0.29-0.95) | 0.034 |
| Non-vertebral fracture | ELD (0.75 μg/day) versus vitamin D 400 IU/day + Ca 610 mg/day | Sakai 2015 | 0.09 (0.01-1.13) | 0.062 |
|  | ELD (0.75 μg/day) versus ALF (1.0 μg/day) | Matsumoto 2011, Jiang 2019 | 0.44 (0.06-3.05) | 0.405 |
| Self-reported falling | ELD (0.75 μg/day) versus vitamin D 400 IU/day + Ca 610 mg/day | Sakai 2015 | 0.78 (0.43-1.42) | 0.415 |

ELD: Eldecalcitol; ALF: Alfacalcidol

**Supplementary Table 2. Summary of other outcomes by different interventions**

| Outcomes | Interventions | Included study | WMD (95% CI) | P value |
| --- | --- | --- | --- | --- |
| FN−BMD (after intervention) | ELD (0.75 μg/day) versus minodronate (50 mg/28 days) and ALF (1.0 μg/day) | Nakatoh 2017 | 0.00 (-0.04 to 0.04) | 1.000 |
| FN−BMD (after intervention) | ELD (0.75 μg/day) versus raloxifene (60 mg/day) and ALF (1.0 μg/day) | Nakatoh 2017 | -0.01 (-0.06 to 0.03) | 0.663 |
| Lumbar BMD (after intervention) | ELD (0.75 μg/day) versus minodronate (50 mg/28 days) and ALF (1.0 μg/day) | Nakatoh 2017 | 0.00 (-0.07 to 0.08) | 1.000 |
| Lumbar BMD (after intervention) | ELD (0.75 μg/day) versus raloxifene (60 mg/day) and ALF (1.0 μg/day) | Nakatoh 2017 | 0.00 (-0.07 to 0.07) | 1.000 |
| BALP (percentage change) | ELD (0.75 μg/day) versus ALF (1.0 μg/day) | Jiang et al | -15.40% (-20.30 to -10.60) | < 0.001 |
| BALP (after intervention) | ELD (0.75 μg/day) versus minodronate (50 mg/28 days) and ALF (1.0 μg/day) | Nakatoh 2017 | 0.50 (-0.76 to 1.76) | 0.437 |
| BALP (after intervention) | ELD (0.75 μg/day) versus raloxifene (60 mg/day) and ALF (1.0 μg/day) | Nakatoh 2017 | 0.70 (-0.57 to 1.97) | 0.280 |
| TRACP−5b (after intervention) | ELD (0.75 μg/day) versus minodronate (50 mg/28 days) and ALF (1.0 μg/day) | Nakatoh 2017 | 84.00 (29.28 to 138.72) | 0.003 |
| TRACP−5b (after intervention) | ELD (0.75 μg/day) versus raloxifene (60 mg/day) and ALF (1.0 μg/day) | Nakatoh 2017 | 57.00 (-0.74 to 114.74) | 0.053 |
| CTX (percentage change) | ELD (0.75 μg/day) versus ALF (1.0 μg/day) | Jiang et al | -38.50% (-50.00 to -27.10) | < 0.001 |
| NTX (percentage change) | ELD (0.75 μg/day) versus ALF (1.0 μg/day) | Uenishi 2018 | -6.70% (-16.10 to 2.70) | 0.162 |
| NTX (percentage change) | ELD (0.75 μg/day) versus plain vitamin D3 (800 IU/day) | Uenishi 2018 | -9.30% (-17.03 to -1.57) | 0.018 |
| NTX (percentage change) | ELD (0.75 μg/day) versus placebo | Uenishi 2018 | -12.40% (-21.28 to -3.52) | 0.006 |
| FCA (after intervention) | ELD (0.75 μg/day) versus ALF (1.0 μg/day) | Uenishi 2018 | 3.50 (-3.40 to 10.40) | 0.320 |
| FCA (after intervention) | ELD (0.75 μg/day) versus plain vitamin D3 (800 IU/day) | Uenishi 2018 | 9.00 (2.88 to 15.12) | 0.004 |
| FCA (after intervention) | ELD (0.75 μg/day) versus placebo | Uenishi 2018 | 13.30 (8.27 to 18.33) | <0.001 |
| Abs (after intervention) | ELD (0.75 μg/day) versus ALF (1.0 μg/day) | Uenishi 2018 | 25.10 (-24.84 to 75.04) | 0.325 |
| Abs (after intervention) | ELD (0.75 μg/day) versus plain vitamin D3 (800 IU/day) | Uenishi 2018 | 65.10 (20.78 to 109.42) | 0.004 |
| Abs (after intervention) | ELD (0.75 μg/day) versus placebo | Uenishi 2018 | 96.20 (59.89 to 132.51) | <0.001 |
| U−Ca (after intervention) | ELD (0.75 μg/day) versus ALF (1.0 μg/day) | Uenishi 2018 | -33.00 (-100.76 to 34.76) | 0.340 |
| U−Ca (after intervention) | ELD (0.75 μg/day) versus plain vitamin D3 (800 IU/day) | Uenishi 2018 | 35.40 (-27.99 to 98.79) | 0.274 |
| U−Ca (after intervention) | ELD (0.75 μg/day) versus placebo | Uenishi 2018 | 87.00 (29.43 to 144.57) | 0.003 |
| 25(OH)D (after intervention) | ELD (0.75 μg/day) versus ALF (1.0 μg/day) | Matsumoto 2011, Uenishi 2018 | -0.80 (-7.45 to 5.86) | 0.814 |
| 25(OH)D (after intervention) | ELD (0.75 μg/day) versus plain vitamin D3 (800 IU/day) | Uenishi 2018 | -10.00 (-17.52 to -2.48) | 0.009 |
| 25(OH)D (after intervention) | ELD (0.75 μg/day) versus placebo | Uenishi 2018 | 3.00 (-5.59 to 11.59) | 0.494 |
| Serum 1,25 (OH)2D (after intervention) | ELD (0.75 μg/day) versus ALF (1.0 μg/day) | Matsumoto 2011 | -72.30 (-76.60 to -68.00) | < 0.001 |
| Serum intact PTH (after intervention) | ELD (0.75 μg/day) versus ALF (1.0 μg/day) | Uenishi 2018 | 9.00 (-4.89 to 22.89) | 0.204 |
| Serum intact PTH (after intervention) | ELD (0.75 μg/day) versus plain vitamin D3 (800 IU/day) | Uenishi 2018 | 4.00 (-7.79 to 15.79) | 0.506 |
| Serum intact PTH (after intervention) | ELD (0.75 μg/day) versus placebo | Uenishi 2018 | 4.00 (-7.41 to 15.41) | 0.492 |
| Serum phosphorus (after intervention) | ELD (0.75 μg/day) versus ALF (1.0 μg/day) | Uenishi 2018 | 0.10 (-0.27 to 0.47) | 0.596 |
| Serum phosphorus (after intervention) | ELD (0.75 μg/day) versus minodronate (50 mg/28 days) and ALF (1.0 μg/day) | Nakatoh 2017 | -0.10 (-0.30 to 0.10) | 0.327 |
| Serum phosphorus (after intervention) | ELD (0.75 μg/day) versus raloxifene (60 mg/day) and ALF (1.0 μg/day) | Nakatoh 2017 | -0.10 (-0.30 to 0.10) | 0.327 |
| Serum phosphorus (after intervention) | ELD (0.75 μg/day) versus plain vitamin D3 (800 IU/day) | Uenishi 2018 | 0.00 (-0.32 to 0.32) | 1.000 |
| Serum phosphorus (after intervention) | ELD (0.75 μg/day) versus placebo | Uenishi 2018 | 0.08 (-0.01 to 0.17) | 0.081 |
| Urinary phosphorus (after intervention) | ELD (0.75 μg/day) versus ALF (1.0 μg/day) | Uenishi 2018 | 0.06 (-0.03 to 0.15) | 0.191 |
| Urinary phosphorus (after intervention) | ELD (0.75 μg/day) versus plain vitamin D3 (800 IU/day) | Uenishi 2018 | 0.06 (-0.02 to 0.14) | 0.142 |
| Urinary phosphorus (after intervention) | ELD (0.75 μg/day) versus placebo | Uenishi 2018 | 0.08 (-0.01 to 0.17) | 0.081 |
| Serum calcium (after intervention) | ELD (0.75 μg/day) versus ALF (1.0 μg/day) | Uenishi 2018 | 0.00 (-0.24 to 0.24) | 1.000 |
| Serum calcium (after intervention) | ELD (0.75 μg/day) versus minodronate (50 mg/28 days) and ALF (1.0 μg/day) | Nakatoh 2017 | 0.20 (-0.05 to 0.45) | 0.117 |
| Serum calcium (after intervention) | ELD (0.75 μg/day) versus raloxifene (60 mg/day) and ALF (1.0 μg/day) | Nakatoh 2017 | 0.20 (-0.05 to 0.45) | 0.117 |
| Serum calcium (after intervention) | ELD (0.75 μg/day) versus plain vitamin D3 (800 IU/day) | Uenishi 2018 | 0.10 (-0.08 to 0.28) | 0.276 |
| Serum calcium (after intervention) | ELD (0.75 μg/day) versus placebo | Uenishi 2018 | 0.05 (0.02 to 0.08) | 0.001 |

BALP: bone-specific alkaline phosphatase; CTX: serum type I collagen C-telopeptide; NTX: amino terminal peptide; FCA: fractional calcium absorption; Abs: absorbed calcium; U-Ca: urinary excretion of calcium; ELD: Eldecalcitol; ALF: Alfacalcidol

**Supplementary Table 3. Summary of adverse events by different interventions**

| Outcomes | Interventions | Included studies | OR (95% CI) | P value |
| --- | --- | --- | --- | --- |
| Discontinued treatment | ELD versus placebo | Matsumoto 2005 | 1.77 (0.58-5.42) | 0.315 |
|  | ELD versus ALF | Matsumoto 2011, Jiang 2019 | 0.66 (0.36-1.20) | 0.172 |
| Any adverse events | ELD versus vitamin D + Ca | Saito 2015 | 1.01 (0.47-2.08) | 0.979 |
|  | ELD versus ALF | Matsumoto 2011, Jiang 2019 | 1.04 (0.56-1.93) | 0.898 |
| Any serious events | ELD versus placebo | Matsumoto 2005 | 1.28 (0.41-3.99) | 0.674 |
|  | ELD versus ALF | Matsumoto 2011, Jiang 2019 | 0.90 (0.63-1.28) | 0.548 |
| **Urinary and Reproductive system disorders** |  |  |  |  |
| Urinary tract infection | ELD versus ALF | Jiang 2019 | 0.78 (0.34-1.82) | 0.562 |
| Renal and urinary disorders | ELD versus vitamin D + Ca | Saito 2015 | 0.20 (0.01-4.18) | 0.298 |
|  | ELD versus ALF | Jiang 2019 | 0.81 (0.26-2.48) | 0.712 |
| Cystitis | ELD versus placebo | Matsumoto 2005 | 3.51 (0.44-27.84) | 0.234 |
|  | ELD versus ALF | Matsumoto 2011 | 0.82 (0.52-1.29) | 0.380 |
| Urolithiasis | ELD versus ALF | Matsumoto 2011 | 1.40 (0.44-4.44) | 0.568 |
| Urine calcium increased | ELD versus placebo | Matsumoto 2005 | 7.34 (0.97-55.68) | 0.054 |
|  | ELD versus ALF | Matsumoto 2011, Jiang 2019 | 1.64 (1.22-2.20) | 0.001 |
| Reproductive system and breast disorders | ELD versus vitamin D + Ca | Saito 2015 | 0.99 (0.06-16.04) | 0.994 |
| **Locomotor system disorders** |  |  |  |  |
| Musculoskeletal and connective tissue disorders | ELD versus vitamin D + Ca | Saito 2015 | 0.87 (0.49-1.54) | 0.630 |
|  | ELD versus ALF | Jiang 2019 | 3.44 (0.70-16.91) | 0.128 |
| Back pain | ELD versus ALF | Matsumoto 2011, Jiang 2019 | 0.90 (0.65-1.23) | 0.517 |
| Osteoarthritis | ELD versus ALF | Matsumoto 2011 | 1.20 (0.85-1.69) | 0.298 |
| Arthralgia | ELD versus ALF | Matsumoto 2011, Jiang 2019 | 1.07 (0.74-1.55) | 0.720 |
| Periarthritis | ELD versus ALF | Matsumoto 2011 | 1.10 (0.71-1.71) | 0.671 |
| Joint sprain | ELD versus ALF | Matsumoto 2011 | 1.03 (0.61-1.76) | 0.913 |
| Spinal osteoarthritis | ELD versus ALF | Matsumoto 2011 | 0.66 (0.38-1.13) | 0.135 |
| Pain in extremity | ELD versus ALF | Matsumoto 2011, Jiang 2019 | 0.91 (0.59-1.42) | 0.674 |
| Contusion | ELD versus ALF | Matsumoto 2011 | 1.01 (0.75-1.34) | 0.946 |
| **Digestive system disorders** |  |  |  |  |
| Gastrointestinal disorders | ELD versus vitamin D + Ca | Saito 2015 | 0.94 (0.52-1.72) | 0.839 |
| Gastroenteritis | ELD versus ALF | Matsumoto 2011 | 1.13 (0.75-1.75) | 0.584 |
| Abdominal distension | ELD versus ALF | Jiang 2019 | 1.55 (0.49-4.87) | 0.454 |
| Gastritis | ELD versus ALF | Matsumoto 2011, Jiang 2019 | 1.01 (0.65-1.56) | 0.964 |
| Stomach discomfort | ELD versus ALF | Matsumoto 2011 | 1.08 (0.62-1.89) | 0.787 |
| Stomachache | ELD versus placebo | Matsumoto 2005 | 3.53 (0.19-64.97) | 0.395 |
|  | ELD versus ALF | Matsumoto 2011 | 1.15 (0.71-1.88) | 0.570 |
| Constipation | ELD versus ALF | Matsumoto 2011 | 0.64 (0.42-0.98) | 0.039 |
| Hepatobiliary disorders | ELD versus vitamin D + Ca | Saito 2015 | 0.33 (0.01-8.12) | 0.517 |
| Diarrhea | ELD versus ALF | Matsumoto 2011, Jiang 2019 | 1.17 (0.78-1.75) | 0.446 |
| **Nervous system disorders** |  |  |  |  |
| Nervous system disorders | ELD versus vitamin D + Ca | Saito 2015 | 1.14 (0.40-3.27) | 0.807 |
| **Respiratory system disorders** |  |  |  |  |
| Upper respiratory tract infection | ELD versus ALF | Jiang 2019 | 0.77 (0.43-1.40) | 0.385 |
| Respiratory, thoracic and mediastinal disorders | ELD versus vitamin D + Ca | Saito 2015 | 0.86 (0.30-2.45) | 0.778 |
| Cough | ELD versus ALF | Jiang 2019 | 0.84 (0.33-2.14) | 0.715 |
| **Circulatory system disorders** |  |  |  |  |
| Vascular disorders | ELD versus vitamin D + Ca | Saito 2015 | 2.55 (0.48-13.42) | 0.271 |
| Cardiac disorders | ELD versus vitamin D + Ca | Saito 2015 | 3.03 (0.31-29.57) | 0.340 |
| Blood and lymphatic system disorders | ELD versus vitamin D + Ca | Saito 2015 | 0.49 (0.04-5.49) | 0.570 |
| Immune system disorders | ELD versus vitamin D + Ca | Saito 2015 | 3.00 (0.12-74.45) | 0.503 |
| Hypertension | ELD versus ALF | Matsumoto 2011 | 1.15 (0.72-1.82) | 0.555 |
| Blood calcium increased | ELD versus placebo | Matsumoto 2005 | 13.17 (0.78-221.52) | 0.073 |
|  | ELD versus ALF | Matsumoto 2011, Jiang 2019 | 1.17 (0.55-2.52) | 0.682 |
| **Other disorders** |  |  |  |  |
| Infection | ELD versus vitamin D + Ca | Saito 2015 | 1.38 (0.81-2.37) | 0.240 |
| General disorders and administration site conditions | ELD versus vitamin D + Ca | Saito 2015 | 0.16 (0.02-1.33) | 0.087 |
| Injury, poisoning, and procedural complications | ELD versus vitamin D + Ca | Saito 2015 | 0.81 (0.47-1.40) | 0.449 |
| Skin and subcutaneous tissue disorders | ELD versus vitamin D + Ca | Saito 2015 | 0.80 (0.38-1.68) | 0.556 |
| Exanthem | ELD versus ALF | Matsumoto 2011 | 0.44 (0.23-0.81) | 0.011 |
| Eczema | ELD versus ALF | Matsumoto 2011 | 1.08 (0.72-1.63) | 0.712 |
| Ear and labyrinth disorders | ELD versus vitamin D + Ca | Saito 2015 | 8.47 (1.04-68.92) | 0.046 |
| Eye disorders | ELD versus vitamin D + Ca | Saito 2015 | 0.42 (0.14-1.26) | 0.122 |
| Conjunctivitis | ELD versus placebo | Matsumoto 2005 | 3.64 (0.20-66.99) | 0.384 |
| Nasopharyngitis | ELD versus ALF | Matsumoto 2011, Jiang 2019 | 1.09 (0.88-1.36) | 0.438 |
| Metabolism and nutrition disorders | ELD versus vitamin D + Ca | Saito 2015 | 0.74 (0.16-3.37) | 0.699 |
| Psychiatric disorders | ELD versus vitamin D + Ca | Saito 2015 | 0.99 (0.14-7.16) | 0.992 |
| Nausea | ELD versus ALF | Jiang 2019 | 1.69 (0.48-5.93) | 0.413 |
| Toothache | ELD versus ALF | Jiang 2019 | 3.44 (0.70-16.91) | 0.128 |
| Dermatitis contact | ELD versus ALF | Matsumoto 2011 | 1.38 (0.83-2.28) | 0.212 |
| Insomnia | ELD versus ALF | Matsumoto 2011 | 1.06 (0.65-1.75) | 0.818 |
| Dizziness | ELD versus ALF | Matsumoto 2011, Jiang 2019 | 0.84 (0.53-1.32) | 0.454 |
| Cataract | ELD versus ALF | Matsumoto 2011 | 1.19 (0.70-2.02) | 0.520 |
| Death | ELD versus ALF | Matsumoto 2011 | 0.66 (0.19-2.36) | 0.518 |
| Cancer | ELD versus vitamin D + Ca | Saito 2015 | 1.32 (0.29-6.04) | 0.719 |
|  | ELD versus ALF | Matsumoto 2011 | 0.68 (0.31-1.49) | 0.340 |
| Headache | ELD versus placebo | Matsumoto 2005 | 4.82 (0.27-85.80) | 0.284 |
|  | ELD versus ALF | Matsumoto 2011 | 1.30 (0.84-2.01) | 0.232 |

ELD: Eldecalcitol; ALF: Alfacalcidol
